# Современные взгляды на лечение инсулиномы

**DOI:** 10.14341/probl13281

**Published:** 2024-02-28

**Authors:** Т. М. Черных, Д. А. Малюгин, М. В. Хачатуров, А. А. Шефер, В. И. Золоедов

**Affiliations:** Воронежский государственный медицинский университет им. Н.Н. Бурденко; Воронежский государственный медицинский университет им. Н.Н. Бурденко; Первый московский государственный медицинский университет им. И.М. Сеченова; Воронежский государственный медицинский университет им. Н.Н. Бурденко; Воронежский государственный медицинский университет им. Н.Н. Бурденко

**Keywords:** инсулинома, хирургическое лечение, медикаментозное лечение, таргетная терапия

## Abstract

**Актуальность:**

Актуальность. Инсулинома является наиболее часто встречающейся гормонально активной нейроэндокринной опухолью (НЭО) поджелудочной железы. В последние годы отмечается тенденция к росту заболеваемости НЭО, в частности инсулиномой.

**Цель:**

Цель. Обобщение и анализ текущих данных о различных подходах к лечению инсулиномы. Наш обзор включает комплексную оценку достоинств и недостатков доступных на сегодняшний день методов лечения инсулиномы в сравнении с опытом прошлых лет, а также обзор перспективных методов, не получивших на данный момент широкого распространения.

**Материалы и методы:**

Материалы и методы. Анализ литературы из таких баз данных как: научная электронная библиотека elibrary.ru, Pubmed, Google Scholar, MedLine, Scopus и Web of Science.

**Результаты:**

Результаты. Наиболее часто для лечения инсулиномы применяется хирургическое лечение. Для пациентов с высоким операционным риском могут применяться альтернативные методы, такие как абляция алкоголем, радиочастотная абляция, эмболизация опухоли. Медикаментозное лечение включает применение аналогов соматостатина, диазоксида. В литературе описывается потенциальная польза от применения бета-адреноблокаторов, фенитоина, глюкагона, однако в клинических исследованиях указанные препараты не продемонстрировали значимого эффекта. Для лечения злокачественной и метастатически распространенной инсулиномы применяется таргетная терапия (прежде всего эверолимус), химиотерапия, а также эмболизация (в том числе химиоэмболизация, радиоэмболизация), радиочастотная абляция (РЧА), микроволновая абляция и криоабляция, ультразвуковая абляция (HIFU), лазерная абляция, брахитерапия, необратимая электропорация.

**Заключение:**

Заключение. Изучение новых препаратов остается важной задачей ученых, среди них наиболее перспективными являются новые поколения аналогов соматостатина, таргетные и химиотерапевтические препараты. Инсулинома — редкая НЭО, что обуславливает трудности для проведения рандомизированных контролируемых испытаний и проспективных исследований. Именно поэтому практикующим врачам и ученым необходимо поддерживать тесный контакт и учитывать опыт лечения каждого пациента, что поможет в будущем разрабатывать эффективные лечебные алгоритмы.

## Abstract (en)

**Relevance:**

Relevance: Insulinoma is the most common hormonally active neuroendocrine tumor (NET) of the pancreas. In recent years, there has been a trend towards an increase in the incidence of NET especially insulinoma.

**Aim:**

Aim: Summarizing and analyzing current data on various approaches to the treatment of insulinoma. Our review includes a comprehensive assessment of the advantages and disadvantages of currently available insulinoma treatment methods in comparison with past experience, as well as a review of promising methods that are not currently widely used.

**Materials and methods:**

Materials and methods: Analysis of literature from such databases as scientific electronic library elibrary.ru, Pubmed, Google Scholar, MedLine, Scopus and Web of Science.

**Results:**

Results: The most common treatment for insulinoma is surgery. For patients with high operative risk, alternative methods such as alcohol ablation, radiofrequency ablation, and tumor embolization may be used. Medications include the use of somatostatin analogues, diazoxide. The literature describes the potential benefit of the use of beta-blockers, phenytoin, glucagon, however, in clinical trials, these drugs have not demonstrated a significant effect. For the treatment of malignant and metastatically advanced insulinoma, targeted therapy (primarily Everolimus), chemotherapy, as well as embolization (including chemoembolization, radioembolization), radiofrequency ablation (RFA), microwave ablation and cryoablation, ultrasound ablation (HIFU), laser ablation, brachytherapy, irreversible electroporation are used.

**Conclusion:**

Conclusion: The study of new drugs is an important task for scientists, among medications the most promising are new generations of somatostatin analogues, targeted drugs and chemotherapy drugs. The rare frequency of insulinoma makes it difficult to conduct randomized controlled trials and prospective studies. That is why physicians and scientists need to maintain close contacts with each other and take into account the experience of treating each patient with such disease, which will help develop effective treatment algorithms in the future.

## ВВЕДЕНИЕ

Нейроэндокринные опухоли (НЭО) являются гетерогенной группой новообразований, происходящих из нейроэндокринных клеток эмбриональной кишки, обладающих биологически активными свойствами. Наиболее часто НЭО локализуются в желудочно-кишечном тракте (66%) [1]. НЭО относятся к редким видам опухолей и не всегда сопровождаются возникновением клинической симптоматики, вследствие чего сведения об их эпидемиологии весьма ограничены. Согласно данным Национального онкологического института США, в 2004 г. показатель вновь выявленных нейроэндокринных неоплазий составил 5,25 на 100 000 населения, заметно увеличившись по сравнению с 1973 г. (1 на 100 000 населения), а распространенность нейроэндокринных неоплазий составила 35 на 100 000 населения [2].

Особый интерес представляют функционально активные НЭО, поскольку гиперпродукция гормонов может приводить к выраженным симптомам, значительно ухудшающим состояние пациента.

Инсулинома является наиболее распространенной НЭО поджелудочной железы (ПЖ) [3]. Согласно данным эпидемиологического исследования, среди 229 пациентов с НЭО ПЖ выявлено 48 пациентов с функционально активными НЭО, среди которых доля пациентов с инсулиномой составила 56,3%, с гастриномой — 25%, c глюкагономой — 4,2%, с другими НЭО — 14,9% [4].

Инсулинома — это тип функциональной НЭО, проявляющийся гипогликемией, вызванной неадекватно высокой секрецией инсулина. Заболеваемость инсулиномой составляет 1–4 случая на миллион человек в год [5]. Наиболее часто инсулинома представляет собой одиночную доброкачественную опухоль, однако в 5,8% случаев инсулинома является злокачественной, а в 6–7,6% ассоциирована с синдромом множественной эндокринной неоплазии 1 типа (МЭН-1) [6][7].

Основным клиническим симптомом инсулиномы является гипогликемия натощак, встречающаяся у 73% пациентов. Около 20% пациентов отмечают симптомы гипогликемии как натощак, так и после приема пищи. Более того, в последние годы выявлена тенденция к увеличению числа пациентов, единственной жалобой которых является постпрандиальная гипогликемия. У большинства пациентов отмечается увеличение массы тела [7].

Проявления гипогликемии вариабельны. Они могут включать: чувство голода, симптомы активации симпатоадреналовой системы, в т.ч. сердцебиение, дрожь, потоотделение, приступы паники, а также нейрогликопенические симптомы, такие как нечеткость зрения, спутанность сознания, судороги, изменение поведения, амнезия гипогликемического эпизода [8]. Совокупность симптомов инсулиномы относится к понятию «триада Уиппла» — симптомы гипогликемии, подтвержденная низкая концентрация глюкозы в крови, улучшение самочувствия при приеме продуктов, содержащих глюкозу. Клинические проявления инсулиномы представлены на рис. 1.

**Figure:**
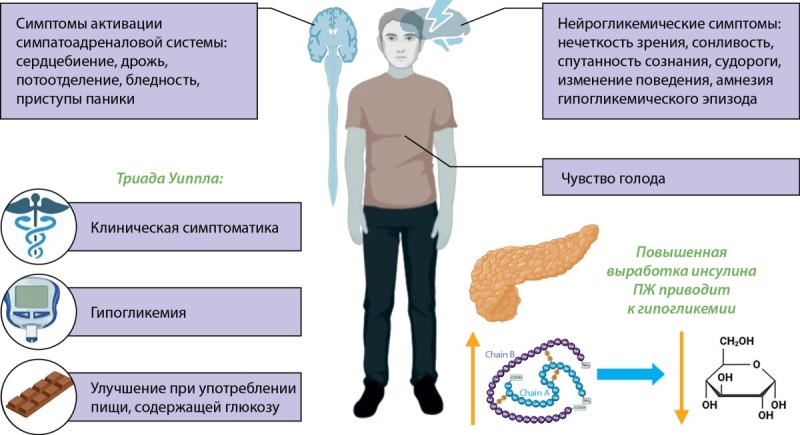
Рисунок 1. Клинические проявления инсулиномы. ПЖ — поджелудочная железа.

Целью нашего исследования является обобщение и анализ текущих данных о различных подходах к лечению инсулиномы. Обзор включает комплексную информацию о доступных на сегодняшний день методах лечения (как хирургических, так и нехирургических) данного вида НЭО в сравнении с опытом прошлых лет, а также о перспективных методах лечения, пока не получивших широкого распространения.

## МАТЕРИАЛЫ И МЕТОДЫ

С использованием ключевых слов «insulinoma», «treatment», «surgical treatment», «drug treatment», «target therapy» и их аналогов на русском языке нами выбрана и проанализирована литература из таких баз данных, как: научная электронная библиотека elibrary.ru, Pubmed, Google Scholar, MedLine, Scopus и Web of Science. Статьи выбирались на основании значимости для понимания текущего состояния проблемы лечения инсулиномы в сравнении с опытом прошлых лет, а также перспектив в лечении данного вида НЭО. Изучены аннотации и полнотекстовые версии публикаций. Для анализа отобрано 60 статей за период с 1973 по 2022 годы, среди которых 26 статей — за последние пять лет (2017–2022 гг).

## ХИРУРГИЧЕСКОЕ ЛЕЧЕНИЕ

Для лечения инсулиномы используется в первую очередь оперативное лечение, после которого в большинстве случаев отмечается выздоровление и отсутствие рецидивов.

При доброкачественных инсулиномах рекомендуется хирургическое лечение, независимо от локализации опухоли. Выбор процедуры (лапароскопическая или открытая операция) зависит прежде всего от размеров и локализации опухоли. Лапароскопическая резекция может быть осуществлена для небольших инсулином (размерами до 2 см на момент постановки диагноза); инсулином, расположенных в теле или хвосте ПЖ [9]; при близости опухоли к протоку ПЖ предпочтительной тактикой является открытое вмешательство [10].

Следует отметить, что частота образования послеоперационных свищей ПЖ составляет в среднем около 21% и не отличается при лапароскопическом вмешательстве и открытом, при этом лапароскопическая операция имеет в качестве преимущества более короткое послеоперационное пребывание пациента в стационаре (по данным R. Naples и соавт.: 4 дня против 7 дней) [11].

Тип оперативного вмешательства также определяется рядом особенностей, прежде всего — биологической агрессивностью опухоли и ее локализацией. Частичная резекция (резекция головки ПЖ, дистальная резекция с сохранением селезенки) показана в случае, если опухоль прилежит к протоку ПЖ. При высокодифференцированных инсулиномах G1 и G2 операцией выбора является энуклеация («вылущивание» опухоли) [12]. Выполнение энуклеации или частичной резекции обеспечивает максимальное сохранение паренхимы ПЖ, что снижает риск экзокринной и эндокринной недостаточности [10]. Радикальную резекцию (например, резекция хвоста и тела ПЖ различного объема, в том числе с удалением селезенки) следует рассматривать для пациентов, у которых поражение не одиночное, плохо капсулированное, более 4 см в диаметре и затрагивает главный панкреатический проток или находится рядом с ним [10].

При резектабельной злокачественной инсулиноме возможны различные варианты оперативного лечения, в т.ч.: удаление первичной опухоли с регионарной лимфодиссекцией, циторедуктивные операции, комбинированные и сочетанные операции, резекция или трансплантация печени при печеночных метастазах [1][10].

Особую категорию пациентов составляют беременные женщины. У данного контингента инсулинома наиболее часто диагностируется в первом триместре беременности, при этом оперативное вмешательство рекомендуется выполнять во втором триместре либо после родов [13].

## МАЛОИНВАЗИВНЫЕ МЕТОДЫ ЛЕЧЕНИЯ

К альтернативным методам лечения инсулиномы можно отнести алкогольную абляцию под контролем эндоскопического УЗИ, радиочастотную абляцию, эмболизацию опухоли. Такие методы лечения могут быть предложены в качестве альтернативы пациентам, отказывающимся от операции, пожилым людям, пациентам с плохим общим состоянием, пациентам с множественными абдоминальными операциями в анамнезе и тем, у кого повышен риск периоперационных осложнений по другим причинам.

В 2006 г. опубликованы результаты лечения с помощью абляции этанолом [14]. Пациентка, 78 лет, находилась в тяжелом состоянии, в связи с чем принято решение отказаться от оперативного вмешательства. В качестве альтернативы выбрана деструкция этанолом — в опухоль введено в общей сложности 8 мл 95-процентного этанола. Авторы заявили о достижении стойкой клинической и биохимической ремиссии и пришли к выводу, что этот метод может быть использован у пациентов с противопоказаниями к хирургическому лечению. Согласно недавним данным, показатель клинического успеха при абляции этанолом НЭО ПЖ составил 87,9% (95% ДИ: 66,2–96,4%), а послеоперационные осложнения возникли у 21,2% испытуемых [15]. Однако в представленном исследовании большинство опухолей ПЖ были гормонально неактивными. W. Paik и соавт. рассмотрели эффективность и безопасность абляции этанолом НЭО. В данном исследовании доля пациентов с инсулиномой составила 3 из 8 пациентов. Из 8 пациентов не удалось достичь ремиссии у двух пациентов — с солидной псевдопапиллярной опухолью ПЖ и с функционально неактивной НЭО, причем у последнего после абляции развился острый панкреатит. У троих пациентов с инсулиномой достигнута ремиссия, у одного из них наблюдалась абдоминальная боль после вмешательства [16].

В 2018 г. S. Qin и соавт. предложили использовать абляцию лауромакраголом (препарат повреждает эндотелий, вызывая коагуляционный некроз, что приводит к склерозированию сосудов) под контролем УЗИ как метод, сопровождающийся меньшим риском возникновения побочных эффектов по сравнению с оперативным вмешательством и абляцией этанолом. Абляция лауромакраголом под УЗИ-контролем выполнена у 7 пациентов, при этом у всех после процедуры отмечалось улучшение самочувствия и биохимическая ремиссия, побочные эффекты отсутствовали [17].

Радиочастотная абляция (РЧА) является еще одним методом, который применяется для лечения опухолей ПЖ, в частности нейроэндокринных. Согласно данным метаанализа S. Fegrachi и соавт., эффективность РЧА при местно-распространенном раке ПЖ сопоставима с результатами оперативного вмешательства с последующей химиотерапией [18]. Лечение инсулиномы с помощью РЧА сопровождается регрессом клинической симптоматики и размеров опухоли [19][20]. При проведении РЧА также могут возникать побочные эффекты, например, в описании клинического случая M. Kluz и соавт. у пациента при РЧА инсулиномы возник панкреонекроз, однако впоследствии пациент был успешно вылечен [20].

Другой нехирургической альтернативой может быть эмболизация опухоли. В 2008 г. G. Rott и соавт. сообщили об успешно выполненной эмболизации инсулиномы у 84-летней пациентки. Эмболизация выполнена 2 мл связанных с желатином трисакриловых частиц диаметром 300-500 мкм, разведенных 5 мл контрастного вещества и 10 мл физиологического раствора. В послеоперационном периоде отмечались абдоминальные боли, панкреатит и сахарный диабет, однако все они имели транзиторный характер и впоследствии регрессировали. В течение года наблюдалась клиническая и биохимическая ремиссия без отсроченных осложнений [21]. Селективная эмболизация может быть использована как отдельно, так и в сочетании с внутриартериальной химиотерапией [10].

## МЕДИКАМЕНТОЗНОЕ ЛЕЧЕНИЕ

Одним из важнейших мероприятий лечения инсулиномы является рекомендация для пациента частых дробных приемов пищи с целью предотвращения приступов гипогликемии [22].

Медикаментозное лечение аналогами соматостатина рекомендуется использовать в предоперационном периоде, а также у пациентов, которые не могут быть вылечены хирургическим путем: например, с диффузным заболеванием β-клеток, множественными инсулиномами, нерезектабельной злокачественной инсулиномой; у больных с противопоказаниями к операции и у пациентов, отказывающихся от оперативного вмешательства.

У значительной части пациентов с НЭО гиперсекреция гормонов представляет собой серьезную клиническую проблему. Аналоги соматостатина, которые связываются с соответствующими рецепторами, экспрессируемыми на нейроэндокринных опухолевых клетках, успешно используются для лечения НЭО, поскольку снижают секрецию инсулина и других биологически активных веществ, а также оказывают антипролиферативные/противоопухолевые эффекты [23].

Октреотид ингибирует секрецию гормонов посредством активации подтипов 2 и 5 соматостатиновых рецепторов [24]. Наиболее распространенными побочными эффектами при использовании октреотида являются абдоминальные боли, метеоризм, а также отсроченные осложнения, к которым относятся мальабсорбция и желчнокаменная болезнь [25–27].

Терапию рекомендуется начинать с октреотида короткого действия в дозе 100 мкг 3 раза в день [1], в качестве стартовой терапии возможно использование октреотида длительного действия в дозе по 20–30 мг каждые 4 недели [28]. Начало терапии октреотидом короткого действия может быть использовано для оценки системной переносимости, особенно побочных эффектов со стороны ЖКТ. При необходимости возможно увеличение дозы октреотида до 40–60 мг 1 раз в 28 дней или уменьшение интервалов между введениями аналогов соматостатина до 1 раза в 14–21 день. Для ланреотида рекомендуемая доза составляет 120 мг подкожно каждые 4 недели, при прогрессировании возможно уменьшение интервала между введениями до 2–3 недель или увеличение дозы до 180 мг. Данное лечение проводится до прогрессирования или непереносимой токсичности. При прогрессировании заболевания дозу аналогов соматостатина необходимо увеличить либо сократить интервалы между введениями, второй компонент комбинированной терапии (таргетный препарат, цитостатик, интерферон-α) следует отменить и заменить на другое лечение. После завершения курса комбинированного лекарственного лечения аналоги соматостатина назначаются в качестве поддерживающей терапии на длительный срок [29].

Несмотря на снижение уровня инсулина, ингибирование аналогами соматостатина контринсулярных гормонов (глюкагон и СТГ) может привести к усугублению гипогликемии. Для контроля приступов гипогликемии, особенно для пациентов со злокачественной инсулиномой, может быть полезен непрерывный мониторинг уровня глюкозы в интерстиции [10].

Медикаментозное лечение может осуществляться не только аналогами соматостатина, но и другими препаратами. В частности, с помощью диазоксида в дозе 50–300 мг/сут [22], антигипогликемическое действие которого обусловлено открытием калиевых каналов β-клеток ПЖ, что приводит к снижению секреции инсулина [30–33]. Прием диазоксида следует прекратить как минимум за неделю до хирургического вмешательства из-за риска интраоперационной гипотензии [34][30]. Поскольку диазоксид вызывает задержку натрия, при его приеме могут отмечаться такие побочные эффекты, как периферические отеки, застойная сердечная недостаточность, а также артериальная гипотензия, нарушение функции почек, увеличение массы тела и гипертрихоз [35]. При необходимости применения высоких доз добавление тиазидных диуретиков (например, гидрохлоротиазида) может предотвращать отеки, но следует помнить, что это также усиливает гипергликемический эффект диазоксида и может привести к гипокалиемии [36].

В 2022 г. Gilliaux Q. и соавт. сообщили о клиническом случае перевода пациента с предоперационной терапии диазоксидом на терапию ланреотидом из-за серьезных побочных эффектов — одышки и персистирующей гипогликемии. При этом лечение ланреотидом оказалось высокоэффективным и не сопровождалось возникновением значимых побочных эффектов, что значительно улучшило качество жизни пациента [37].

В исследовании 1997 г. при оценке эффективности диазоксида для коррекции гипогликемии сообщалось, что у 59% пациентов симптомы полностью отсутствовали, а у 38% проявлялись крайне редко, при этом побочные эффекты развивались у 47% больных [30]. В 2019 году также проведено исследование по оценке эффективности и переносимости диазоксида. Препарат продемонстрировал эффективность у 9 пациентов из 20, оказался неэффективен также у 9 пациентов (у двоих эффективность оценить не удалось). Побочные эффекты зарегистрированы у 13 пациентов, 11 из которых потребовалось прекращение лечения диазоксидом в связи с тяжелой тромбоцитопенией либо значительной задержкой жидкости [38]. Таким образом, результаты этих двух исследований демонстрируют, что приблизительно у половины больных диазоксид эффективен, а у другой половины требуется прекращение терапии в связи с непереносимостью. Что касается аналогов соматостатина, их эффективность, по разным данным, составляет от 35 до 50% [34][39], а в одном исследовании с участием 21 пациента препарат был эффективен у 67% обследованных [40]. Разнородность данных, а также малое число пациентов обуславливают отсутствие четкой позиции по вопросу выбора медикаментозной терапии между диазоксидом и аналогами соматостатина, поэтому подход в данном вопросе должен быть строго индивидуальным.

Следует обратить особое внимание на аналог соматостатина второго поколения — пасиреотид. Данный препарат обладает большим сродством к рецепторам соматостатина 1, 2, 3 и 5 типов, чем препараты первого поколения (октреотид и ланреотид), и может быть эффективен при лечении злокачественной инсулиномы [41]. Oziel-Taieb S. и соавт. (2022 г.) сообщили о регрессе симптоматики инсулиномы при применении пасиреотида. В данном клиническом случае пациент имел злокачественную метастатическую инсулиному с рефрактерной гипогликемией, а лечение октреотидом, ланреотидом и диазоксидом не приводило к заметному улучшению состояния [42].

Что касается беременных пациенток, использование медикаментозной терапии необходимо проводить в случае, если ожидаемая польза для матери превышает риск для плода. В качестве препарата первой линии рекомендуется использование диазоксида. В качестве препаратов второй линии, а также при злокачественных инсулиномах используются аналоги соматостатина — в первую очередь октреотид. При злокачественных инсулиномах возможно также применение эверолимуса [13].

Другие лекарственные средства, ингибирующие биосинтез или высвобождение инсулина, также рассматриваются в литературе как потенциальные способы терапии инсулиномы, однако не используются широко в настоящее время и могут быть предложены как альтернативные способы для пациентов с индивидуальной непереносимостью препаратов первой линии.

Антагонисты β-адренорецепторов подавляют секрецию инсулина, следовательно, могут быть полезны при лечении органического гиперинсулинизма. Применение пропранолола было связано со снижением уровня инсулина в плазме и купированием приступов гипогликемии у пациентов с доброкачественной или злокачественной инсулиномой. Однако этот препарат также может маскировать адренергические симптомы гипогликемии и ингибировать мышечный гликогенолиз, что обуславливает риск ухудшения клинической симптоматики. При необходимости приема препарата следует проявлять особую осторожность и тщательно контролировать состояние больного [36]. В прошлом веке сообщалось об эффективности пропранолола у больных с инсулиномой, однако в последние годы этот вопрос активно не изучается в связи с эффективностью у большей части пациентов аналогов соматостатина или диазоксида [43].

Противосудорожный препарат фенитоин ингибирует высвобождение инсулина in vitro из β-клеток. Однако клинически значимый гипергликемический эффект фенитоина отмечается не более чем у одной трети пациентов с инсулиномой. Кроме того, фиксируется высокая частота возникновения побочных эффектов [36].

Применение глюкокортикоидов, усиливающих глюконеогенез и вызывающих инсулинорезистентность, может помочь стабилизировать уровень глюкозы в крови. Рекомендуемая доза преднизолона обычно подбирается индивидуально [36].

Глюкагон может помочь повысить концентрацию глюкозы в крови, но одновременно он способен напрямую стимулировать высвобождение инсулина, что крайне нежелательно [36].

Для лечения злокачественных инсулином возможно применение глюкокортикоидов и таргетной терапии [1]. Что касается последней, для лечения инсулином проведены исследования следующих групп препаратов: ингибиторы киназы mTOR, ингибиторы тирозинкиназы и мультикиназные ингибиторы. Наибольшую эффективность продемонстрировал ингибитор киназы mTOR — эверолимус. У пациентов с неоперабельными или злокачественными инсулиномами эверолимус в дозе 10 мг/сут может нормализовать уровень глюкозы. Ингибиторы mTOR также обладают способностью снижать секрецию инсулина и повышать резистентность к инсулину. Другие таргетные препараты обладают значительно меньшей способностью снижать секрецию инсулина и уменьшать симптомы гипогликемии, демонстрируя на данный момент значительно более скромные результаты [22][36]. 5-азацитидин может быть эффективен для лечения злокачественной инсулиномы. Препарат стимулирует аутофагию клеток инсулиномы в условиях оксидативного стресса, индуцированного перекисью водорода [44]. Однако исследование данного препарата проведено на клеточных линиях инсулиномы, а не на пациентах, поэтому судить о его реальной эффективности пока преждевременно.

Медикаментозные методы лечения инсулиномы представлены на рисунке 2.

**Figure:**
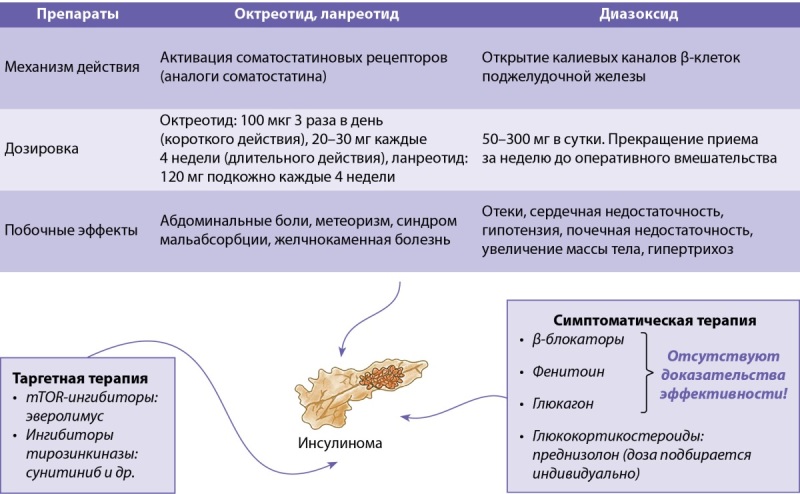
Рисунок 2. Медикаментозные методы лечения инсулиномы.

## КОМПЛЕКСНАЯ ТЕРАПИЯ ЗЛОКАЧЕСТВЕННЫХ, В ТОМ ЧИСЛЕ МЕТАСТАТИЧЕСКИ-РАСПРОСТРАНЕННЫХ ИНСУЛИНОМ

Пациентам, у которых заболевание прогрессирует несмотря на прием аналогов соматостатина и объем опухоли, можно уменьшить методом циторедукции, может быть рекомендовано лечение молекулярно-таргетными препаратами: ингибиторами тирозинкиназы (сунитиниб и др.) и ингибиторами mTOR (эверолимус и др.) [1]. Для пациентов с выраженной симптоматикой из-за большого размера опухоли или с быстро растущими метастазами в качестве начального лечения используется химиотерапия вместе с аналогом соматостатина. В зависимости от степени дифференцировки опухоли применяют разные схемы терапии [1]. При инсулиноме значимым эффектом может обладать сочетание эверолимуса с октреотидом или сунитиниба с октреотидом. В качестве химиотерапии при злокачественных инсулиномах может быть использовано совместное введение стрептозоцина и доксорубицина и/или 5-фторурацила, что уменьшает степень выраженности симптомов у 60–70% пациентов [45]. Многообещающей является также схема FOLFOX, включающая сочетание 5-фторурацила с оксалиплатином. В исследовании, проведенном на 115 пациентах, лучшие показатели выживаемости без прогрессирования, а также наиболее яркий регресс клинической симптоматики отмечались у больных с инсулиномой [46].

Для лечения метастатического заболевания печени при отсутствии диффузного процесса, нарушения функции печени или внепеченочных метастазов (например, легочных, канцероматоза брюшины) применяется хирургический метод, который направлен преимущественно на облегчение симптомов, связанных с гиперсекрецией гормонов [47][48]. Также для лечения первичной опухоли и метастазов в печени при злокачественной инсулиноме могут быть использованы такие методы, как эмболизация (в том числе химиоэмболизация или радиоэмболизация), радиочастотная абляция (РЧА), микроволновая абляция и криоабляция, ультразвуковая абляция (HIFU), лазерная абляция, брахитерапия, необратимая электропорация [36].

Эмболизация печеночных артерий применяется в качестве паллиативной техники как альтернативная методика лекарственной терапии для симптоматических пациентов с нерезектабельными метастазами НЭО в печень [1]. Эмболизация выполняется путем инфузии эмболизирующих агентов (поливиниловый спирт или гель-пена) в печеночную артерию через ангиографический катетер [49], а также химиопрепаратов (применяются отдельно или совместно с другими методами эмболизации), вводимыми через печеночную артерию [50]. Эмболизацию можно выполнить радиоактивными изотопами (например, иттрий-90) [51], которые помечаются стеклянными или полимерными микросферами и избирательно доставляются к опухоли через печеночную артерию. В исследовании Kennedy и соавт. (2008) у 148 пациентов с нерезектабельными метастазами в печень применена радиоэмболизация, при этом у 64% наблюдался объективный ответ [52]. В систематическом обзоре при сравнении показаны одинаковые клиническая эффективность и безопасность эмболизации тремя разными методами (трансартериальная эмболизация, трансартериальная химиоэмболизация, трансартериальная радиоэмболизация) у пациентов с нейроэндокринными метастазами в печень. Частота ответа, измеряемая снижением секреции гормонов или рентгенологической регрессией, при эмболизации обычно превышает 50% [53].

Абляцию с использованием радиочастотных и микроволновых волн или криоагента можно использовать в качестве основного метода лечения нейроэндокринных метастазов в печени или как дополнения к хирургическому методу. Данная процедура менее инвазивна, чем резекция печени или эмболизация печеночной артерии. Абляция также может быть особенно полезна для пациентов с рецидивом внутрипеченочного заболевания, у которых хирургические возможности ограничены из-за предшествующей гепатэктомии. В исследовании Mayo и соавт. 66 из 339 пациентов (19%), получавших направленную на печень терапию, подверглись комбинации резекции и абляции при первом хирургическом вмешательстве [54]. Однако абляция применима только к небольшим поражениям (обычно <3 см), и ее долгосрочная эффективность неясна [55].

Трансплантация печени — довольно противоречивый, хотя и перспективный метод лечения печеночных метастазов НЭО. Число пациентов с изолированным метастатическим заболеванием печени, у которых предпринята попытка трансплантации, относительно невелико, а роль данного метода у пациентов с метастатическими НЭО еще не установлена и остается спорной [56]. Тем не менее трансплантация печени может быть использована у пациентов при множественных метастазах злокачественных НЭО в печени при отсутствии внепеченочных метастазов [57][58].

Пептидная рецепторная радионуклидная терапия (ПРРТ) является еще одним вариантом лечения прогрессирующих НЭО поджелудочной железы, экспрессирующих соматостатиновые рецепторы. Для проведения ПРРТ используются радиофармпрепараты, представляющие собой радиоактивные изотопы, связанные с аналогом соматостатина. Аналог соматостатина связывается с соответствующими рецепторами, что приводит к поглощению опухолью радиоактивного вещества. Симптоматические и радиологические ответы отмечены при лечении функциональных НЭО поджелудочной железы, включая инсулиному, с помощью 177Lu-DOTA-TATE [59]. Роль иммунотерапии ингибиторами иммунных контрольных точек только начинает изучаться у пациентов с высокодифференцированными НЭО. Ранние данные свидетельствуют о том, что антитела к PD-1 обладают минимальной активностью в качестве монотерапии [60]. Обобщенные данные о лечении инсулиномы представлены на рисунке 3.

**Figure:**
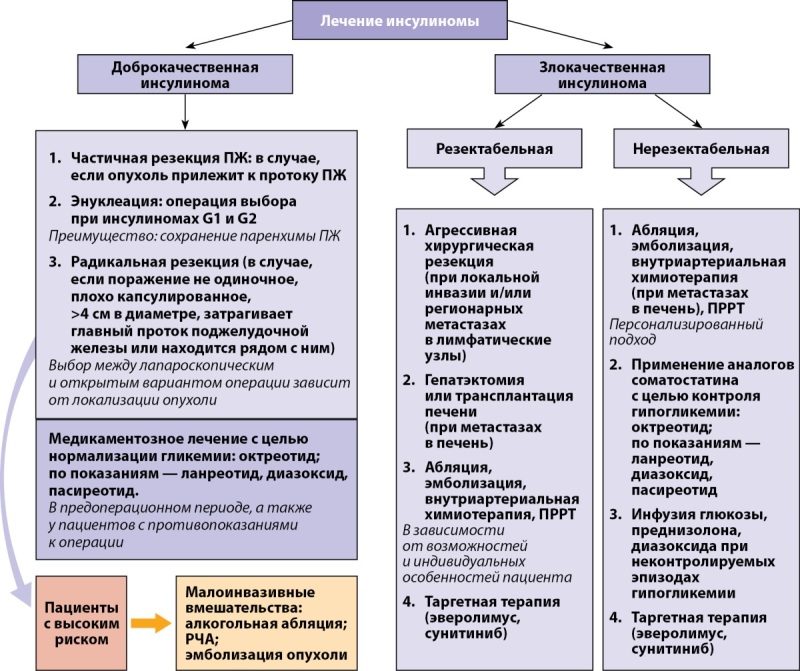
Рисунок 3. Общие принципы лечения инсулиномы.Условные обозначения: ПЖ — поджелудочная железа; РЧА — радиочастотная абляция; ПРРТ — пептидная рецепторная радионуклидная терапия.

## ЗАКЛЮЧЕНИЕ

Проведенный анализ литературы продемонстрировал разнообразие методов лечения инсулиномы, доступных на сегодняшний день. Однако наиболее важным из них остается хирургическое лечение. Выбор лечебной тактики при отсутствии возможности хирургического вмешательства зачастую определяется наличием того или иного оборудования в конкретной медицинской организации. В медикаментозном лечении главная роль отводится аналогам соматостатина и диазоксиду. Изучение новых препаратов остается важной задачей ученых, среди них наиболее перспективными являются новые поколения аналогов соматостатина, таргетные и химиотерапевтические препараты, особенно для лечения злокачественной инсулиномы. Редкая частота данного вида опухоли обуславливает трудности для проведения рандомизированных контролируемых испытаний и проспективных исследований. Именно поэтому практикующим врачам и ученым необходимо поддерживать тесный контакт и учитывать опыт лечения каждого пациента, что поможет в будущем разрабатывать эффективные лечебные алгоритмы.

## ДОПОЛНИТЕЛЬНАЯ ИНФОРМАЦИЯ

Источники финансирования. Работа выполнена по собственной инициативе авторов без финансирования.

Конфликт интересов. Авторы заявляют об отсутствии явного или потенциального конфликта интересов.

Участие авторов. Черных Т.М., Малюгин Д.А., Хачатуров М.В., Шефер А.А. — анализ литературы, написание текста, подготовка рисунков; Золоедов В.И. — научное консультирование и редактирование. Все авторы внесли весомый вклад в написание рукописи, прочитали и одобрили финальную версию.

## References

[cit1] Assotsiatsiya onkologov Rossii, Rossiiskoe obshchestvo klinicheskoi onkologii, Rossiiskaya assotsiatsiya endokrinologov. Federal'nye klinicheskie rekomendatsii po neiroendokrinnym opukholyam. — 2022. — URL: https://oncology-association.ru/wp-content/uploads/2020/09/nejrojendokrijnye_opuholi.pdf

[cit2] Yao James C., Hassan Manal, Phan Alexandria, Dagohoy Cecile, Leary Colleen, Mares Jeannette E., Abdalla Eddie K., Fleming Jason B., Vauthey Jean-Nicolas, Rashid Asif, Evans Douglas B. (2008). One Hundred Years After “Carcinoid”: Epidemiology of and Prognostic Factors for Neuroendocrine Tumors in 35,825 Cases in the United States. Journal of Clinical Oncology.

[cit3] Halfdanarson T.R., Rabe K.G., Rubin J., Petersen G.M. (2008). Pancreatic neuroendocrine tumors (PNETs): incidence, prognosis and recent trend toward improved survival. Annals of Oncology.

[cit4] Muscogiuri Giovanna, Altieri Barbara, Albertelli Manuela, Dotto Andrea, Modica Roberta, Barrea Luigi, Fanciulli Giuseppe, Feola Tiziana, Baldelli Roberto, Ruggeri Rosaria Maddalena, Gallo Marco, Guarnotta Valentina, Malandrino Pasqualino, Messina Erika, Venneri Mary Anna, Giannetta Elisa, Ferone Diego, Colao Annamaria, Faggiano Antongiulio (2020). Epidemiology of pancreatic neuroendocrine neoplasms: a gender perspective. Endocrine.

[cit5] Sotoudehmanesh Rasoul, Hedayat Anooshirvan, Shirazian Nahid, Shahraeeni Shadi, Ainechi Sanaz, Zeinali Fatemeh, Kolahdoozan Shadi (2007). Endoscopic ultrasonography (EUS) in the localization of insulinoma. Endocrine.

[cit6] SERVICE F. JOHN, McMAHON M. MOLLY, O'BRIEN PETER C., BALLARD DAVID J. (2012). Functioning Insulinoma—Incidence, Recurrence, and Long-Term Survival of Patients: A 60-Year Study. Mayo Clinic Proceedings.

[cit7] Placzkowski Kimberly A., Vella Adrian, Thompson Geoffrey B., Grant Clive S., Reading Carl C., Charboneau J. William, Andrews James C., Lloyd Ricardo V., Service F. John (2009). Secular Trends in the Presentation and Management of Functioning Insulinoma at the Mayo Clinic, 1987–2007. The Journal of Clinical Endocrinology & Metabolism.

[cit8] Pratò S, Didonna V, Garletti F, et al. Loss of consciousness in a helicopter pilot as plausible first sign of insulinoma: a case report. Med Lav. 2022; 113(1):e2022007. doi: https://doi.org/10.23749/mdl.v113i1.12037 PMC8902744 35226655

[cit9] España‐Gómez María Nayví, Velázquez‐Fernández David, Bezaury Paulina, Sierra Mauricio, Pantoja Juan Pablo, Herrera Miguel F. (2009). Pancreatic Insulinoma: A Surgical Experience. World Journal of Surgery.

[cit10] Okabayashi Takehiro (2013). Diagnosis and management of insulinoma. World Journal of Gastroenterology.

[cit11] Naples Robert, Thomas Jonah D., Orabi Danny A., Reynolds Jordan P., Robertson Scott, Siperstein Allan E., Walsh R.Matthew, Simon Robert, Shin Joyce J., Jin Judy, Krishnamurthy Vikram D., Berber Eren (2021). A critical analysis of laparoscopic and open approaches to sporadic pancreatic insulinoma resection in the modern era. The American Journal of Surgery.

[cit12] de Carbonnières Anne, Challine Alexandre, Cottereau Anne Ségolène, Coriat Romain, Soyer Philippe, Abou Ali Einas, Prat Frédéric, Terris Benoit, Bertherat Jérôme, Dousset Bertrand, Gaujoux Sébastien (2021). Surgical management of insulinoma over three decades. HPB.

[cit13] Dobrindt Eva M, Mogl Martina, Goretzki Peter E, Pratschke Johann, Dukaczewska Agata K (2021). Insulinoma in pregnancy (a case presentation and systematic review of the literature). Rare Tumors.

[cit14] Jürgensen Christian, Schuppan Detlef, Neser Frank, Ernstberger Jan, Junghans Ulrich, Stölzel Ulrich (2006). EUS-guided alcohol ablation of an insulinoma. Gastrointestinal Endoscopy.

[cit15] Zhang Lu, Tan Shali, Huang Shu, Zhong Chunyu, Lü Muhan, Peng Yan, Tang Xiaowei (2020). The safety and efficacy of endoscopic ultrasound-guided ablation therapy for solid pancreatic tumors: a systematic review. Scandinavian Journal of Gastroenterology.

[cit16] Paik Woo Hyun, Seo Dong Wan, Dhir Vinay, Wang Hsiu-Po (2016). Safety and Efficacy of EUS-Guided Ethanol Ablation for Treating Small Solid Pancreatic Neoplasm. Medicine.

[cit17] Qin Shanyu, Liu Yongru, Ning Hongjian, Tao Lin, Luo Wei, Lu Donghong, Luo Zuojie, Qin Yingfen, Zhou Jia, Chen Junqiang, Jiang Haixing (2017). EUS-guided lauromacrogol ablation of insulinomas: a novel treatment. Scandinavian Journal of Gastroenterology.

[cit18] Fegrachi Samira, Besselink Marc G., van Santvoort Hjalmar C., van Hillegersberg Richard, Molenaar Izaak Quintus (2013). Radiofrequency ablation for unresectable locally advanced pancreatic cancer: a systematic review. HPB.

[cit19] Alyusuf Ebtihal Y., Ekhzaimy Aishah A., Rivera Juan A. (2020). Radiofrequency Ablation as a Primary Therapy for Benign Functioning Insulinoma. AACE Clinical Case Reports.

[cit20] Kluz Michał, Staroń Robert, Krupa Łukasz, Partyka Mariusz, Polkowski Marcin, Gutkowski Krzysztof (2019). Successful endosonography-guided radiofrequency ablation of pancreatic insulinoma. Polish Archives of Internal Medicine.

[cit21] Rott Gernot, Biggemann Martin, Pfohl Martin (2007). Embolization of an Insulinoma of the Pancreas with Trisacryl Gelatin Microspheres as Definitive Treatment. CardioVascular and Interventional Radiology.

[cit22] Giannis D, Moris D, Karachaliou GS, et al. Insulinomas: from diagnosis to treatment. A review of the literature. J BUON. 2020; 25(3):1302-1314 32862570

[cit23] Kulke Matthew H. (2017). Somatostatin Analogues in Neuroendocrine Tumors. Journal of the National Comprehensive Cancer Network.

[cit24] Stueven Anna Kathrin, Kayser Antonin, Wetz Christoph, Amthauer Holger, Wree Alexander, Tacke Frank, Wiedenmann Bertram, Roderburg Christoph, Jann Henning (2019). Somatostatin Analogues in the Treatment of Neuroendocrine Tumors: Past, Present and Future. International Journal of Molecular Sciences.

[cit25] (2018). Update on Surgical Management of Small Bowel Neuroendocrine Tumors. Anticancer Research.

[cit26] Gillis Jane C., Noble Stuart, Goa Karen L. (2009). Octreotide Long-Acting Release (LAR). Drugs.

[cit27] Lamberts Steven W.J., van der Lely Aart-Jan, de Herder Wouter W., Hofland Leo J. (2002). Octreotide. New England Journal of Medicine.

[cit28] Öberg K., Kvols L., Caplin M., Delle Fave G., de Herder W., Rindi G., Ruszniewski P., Woltering E.A., Wiedenmann B. (2004). Consensus report on the use of somatostatin analogs for the management of neuroendocrine tumors of the gastroenteropancreatic system. Annals of Oncology.

[cit29] Orel N.F., Artamonova E.V., Gorbunova V.A., Delektorskaya V.V., i soavt. Prakticheskie rekomendatsii po lekarstvennomu lecheniyu neiroendokrinnykh neoplazii zheludochno-kishechnogo trakta, podzheludochnoi zhelezy i drugikh lokalizatsii. Zlokachestvennye opukholi: Prakticheskie rekomendatsii RUSSCO #3s2, 2021 (tom 11). 30

[cit30] Gill G V, Rauf O, MacFarlane I A (2008). Diazoxide treatment for insulinoma: a national UK survey. Postgraduate Medical Journal.

[cit31] Hirshberg Boaz, Cochran Craig, Skarulis Monica C., Libutti Steven K., Alexander H. Richard, Wood Bradford J., Chang Richard, Kleiner David E., Gorden Phillip (2005). Malignant insulinoma. Cancer.

[cit32] Goode Peter N., Farndon John R., Anderson John, Johnston Ivan D. A., Morte Javier Abascal (2005). Diazoxide in the management of patients with insulinoma. World Journal of Surgery.

[cit33] Ing Roy, Petrakis NicholasL., Ho H.C. (2004). EVIDENCE AGAINST ASSOCIATION BETWEEN WET CERUMEN AND BREAST CANCER. The Lancet.

[cit34] Maggio I., Mollica V., Brighi N., Lamberti G., Manuzzi L., Ricci A. D., Campana D. (2019). The functioning side of the pancreas: a review on insulinomas. Journal of Endocrinological Investigation.

[cit35] Stefanini P, Carboni M, Patrassi N, et al. Beta-islet cell tumors of the pancreas: results of a study on 1,067 cases. Surgery. 1974; 75(4):597-609 4366135

[cit36] de Herder WW, Zandee WT, Hofland J. Insulinoma. In: Feingold KR, Anawalt B, Boyce A, et al., eds. Endotext. South Dartmouth (MA): MDText.com, Inc.; October 25, 2020

[cit37] Gilliaux Quentin, Bertrand Claude, Hanon François, Donckier Julian E. (2020). Preoperative treatment of benign insulinoma: diazoxide or somatostatin analogues?. Acta Chirurgica Belgica.

[cit38] Niitsu Yoshihiro, Minami Isao, Izumiyama Hajime, Hashimoto Koshi, Yoshimoto Takanobu, Satou Fuminori, Tsujino Motoyoshi, Ota Kazuki, Kudo Atsushi, Tanabe Minoru, Yamada Tetsuya, Ogawa Yoshihiro (2018). Clinical outcomes of 20 Japanese patients with insulinoma treated with diazoxide. Endocrine Journal.

[cit39] Ito Tetsuhide, Igarashi Hisato, Jensen Robert T. (2013). Pancreatic neuroendocrine tumors: Clinical features, diagnosis and medical treatment: Advances. Best Practice & Research Clinical Gastroenterology.

[cit40] Vezzosi D., Bennet A., Courbon F., Caron P. (2008). Short‐ and long‐term somatostatin analogue treatment in patients with hypoglycaemia related to endogenous hyperinsulinism. Clinical Endocrinology.

[cit41] Brown Emily, Watkin Daniel, Evans Jonathan, Yip Vincent, Cuthbertson Daniel J. (2017). Multidisciplinary management of refractory insulinomas. Clinical Endocrinology.

[cit42] Oziel-Taieb Sandrine, Maniry-Quellier Jemima, Chanez Brice, Poizat Flora, Ewald Jacques, Niccoli Patricia (2022). Pasireotide for Refractory Hypoglycemia in Malignant Insulinoma- Case Report and Review of the Literature. Frontiers in Endocrinology.

[cit43] Scandellari C., Zaccaria M., De Palo C., Sicolo N., Erle G., Federspil G. (2007). The effect of propranolol on hypoglycaemia. Diabetologia.

[cit44] Filip Kamila, Lewińska Anna, Adamczyk-Grochala Jagoda, Marino Gammazza Antonella, Cappello Francesco, Lauricella Marianna, Wnuk Maciej (2022). 5-Azacytidine Inhibits the Activation of Senescence Program and Promotes Cytotoxic Autophagy during Trdmt1-Mediated Oxidative Stress Response in Insulinoma β-TC-6 Cells. Cells.

[cit45] AlJadir Saadi (2016). Insulinoma: Literature’s Review (2). Endocrinology&Metabolism International Journal.

[cit46] Girot Paul, Baudin Eric, Senellart Hélène, Bouarioua Nadia, Hentic Olivia, Guimbaud Rosine, Walter Thomas, Ferru Aurélie, Roquin Guillaume, Cadiot Guillaume, Pracht Marc, Girot Jean-Baptiste, Malka David, Ducreux Michel, Bennouna Jaafar, Matysiak-Budnik Tamara, Hadoux Julien, Touchefeu Yann (2021). Oxaliplatin and 5-Fluorouracil in Advanced Well-Differentiated Digestive Neuroendocrine Tumors: A Multicenter National Retrospective Study from the French Group of Endocrine Tumors. Neuroendocrinology.

[cit47] Tran Catherine G., Sherman Scott K., Chandrasekharan Chandrikha, Howe James R. (2020). Surgical Management of Neuroendocrine Tumor Liver Metastases. Surgical Oncology Clinics of North America.

[cit48] Qu Yuqing, Li Haoming, Wang Xianling, Chen Yulong, Guo Qinghua, Pei Yu, Du Jin, Dou Jingtao, Ba Jianming, Lv Zhaohui, Mu Yiming (2020). Clinical Characteristics and Management of Functional Pancreatic Neuroendocrine Neoplasms: A Single Institution 20-Year Experience with 286 Patients. International Journal of Endocrinology.

[cit49] Gupta Sanjay, Johnson Marcella M., Murthy Ravi, Ahrar Kamran, Wallace Michael J., Madoff David C., McRae Stephen E., Hicks Marshall E., Rao Sujaya, Vauthey Jean‐Nicolas, Ajani Jaffer A., Yao James C. (2005). Hepatic arterial embolization and chemoembolization for the treatment of patients with metastatic neuroendocrine tumors. Cancer.

[cit50] Christante Dara, Pommier SuEllen, Givi Babak, Pommier Rodney (2008). Hepatic artery chemoinfusion with chemoembolization for neuroendocrine cancer with progressive hepatic metastases despite octreotide therapy. Surgery.

[cit51] Rhee Thomas K., Lewandowski Robert J., Liu David M., Mulcahy Mary F., Takahashi Gary, Hansen Paul D., Benson Al B., Kennedy Andrew S., Omary Reed A., Salem Riad (2008). 90Y Radioembolization for Metastatic Neuroendocrine Liver Tumors. Annals of Surgery.

[cit52] Kennedy Andrew S., Dezarn William A., McNeillie Patrick, Coldwell Doug, Nutting Charles, Carter Dennis, Murthy Ravi, Rose Steven, Warner Richard R. P., Liu David, Palmedo Holger, Overton Carroll, Jones Bonita, Salem Riad (2010). Radioembolization for Unresectable Neuroendocrine Hepatic Metastases Using Resin 90Y-Microspheres: Early Results in 148 Patients. American Journal of Clinical Oncology.

[cit53] Kennedy Andrew, Bester Lourens, Salem Riad, Sharma Ricky A., Parks Rowan W., Ruszniewski Philippe (2014). Role of hepatic intra-arterial therapies in metastatic neuroendocrine tumours (NET): guidelines from the NET-Liver-Metastases Consensus Conference. HPB.

[cit54] Mayo Skye C., de Jong Mechteld C., Pulitano Carlo, Clary Brian M., Reddy Srinevas K., Gamblin T. Clark, Celinksi Scott A., Kooby David A., Staley Charles A., Stokes Jayme B., Chu Carrie K., Ferrero Alessandro, Schulick Richard D., Choti Michael A., Mentha Giles, Strub Jennifer, Bauer Todd W., Adams Reid B., Aldrighetti Luca, Capussotti Lorenzo, Pawlik Timothy M. (2010). Surgical Management of Hepatic Neuroendocrine Tumor Metastasis: Results from an International Multi-Institutional Analysis. Annals of Surgical Oncology.

[cit55] Moug Susan J., Leen Edward, Horgan Paul G., Imrie Clement W. (2008). Radiofrequency Ablation Has a Valuable Therapeutic Role in Metastatic VIPoma. Pancreatology.

[cit56] Gedaly Roberto (2011). Liver Transplantation for the Treatment of Liver Metastases From Neuroendocrine Tumors. Archives of Surgery.

[cit57] Le Treut Y. Patrice, Delpero Jean R., Dousset Bertrand, Cherqui Daniel, Segol Philippe, Mantion Georges, Hannoun Laurent, Benhamou# Guy, Launois Bernard, Boillot Olivier, Domergue Jacques, Bismuth Henri (2003). Results of Liver Transplantation in the Treatment of Metastatic Neuroendocrine Tumors. Annals of Surgery.

[cit58] Olausson Michael, Friman Styrbjörn, Cahlin Christian, Nilsson Ola, Jansson Svante, Wängberg Bo, Ahlman Håkan (2002). Indications and Results of Liver Transplantation in Patients with Neuroendocrine Tumors. World Journal of Surgery.

[cit59] Zandee Wouter T, Brabander Tessa, Blažević Anela, Kam Boen L R, Teunissen Jaap J M, Feelders Richard A, Hofland Johannes, de Herder Wouter W (2019). Symptomatic and Radiological Response to 177Lu-DOTATATE for the Treatment of Functioning Pancreatic Neuroendocrine Tumors. The Journal of Clinical Endocrinology & Metabolism.

[cit60] Strosberg Jonathan, Mizuno Nobumasa, Doi Toshihiko, Grande Enrique, Delord Jean-Pierre, Shapira-Frommer Ronnie, Bergsland Emily, Shah Manisha, Fakih Marwan, Takahashi Shunji, Piha-Paul Sarina A., O'Neil Bert, Thomas Sajeve, Lolkema Martijn P., Chen Menghui, Ibrahim Nageatte, Norwood Kevin, Hadoux Julien (2020). Efficacy and Safety of Pembrolizumab in Previously Treated Advanced Neuroendocrine Tumors: Results From the Phase II KEYNOTE-158 Study. Clinical Cancer Research.

